# The Effectiveness of the KiVa Bullying Prevention Program in Wales, UK: Results from a Pragmatic Cluster Randomized Controlled Trial

**DOI:** 10.1007/s11121-020-01103-9

**Published:** 2020-04-02

**Authors:** Nick Axford, Gretchen Bjornstad, Suzy Clarkson, Obioha C. Ukoumunne, Zoe Wrigley, Justin Matthews, Vashti Berry, Judy Hutchings

**Affiliations:** 1 NIHR ARC South West Peninsula (PenARC), University of Plymouth, Plymouth, UK; 2 University of Exeter Medical School, University of Exeter, Exeter, UK; 3 Bangor University, Bangor, UK; 4 PenARC, University of Exeter, Exeter, UK; 5 University of Cardiff, Cardiff, UK

**Keywords:** Bullying, Prevention, Intervention, Randomized controlled trial, Evidence-based intervention

## Abstract

The study evaluated the implementation fidelity and effectiveness of KiVa, an evidence-based program that aims to prevent and address bullying in schools, with a particular emphasis on changing the role of bystanders. The study was a two-arm waitlist control cluster randomized controlled trial in which 22 primary schools (clusters) (*N* = 3214 students aged 7–11) were allocated using a 1:1 ratio to intervention (KiVa; 11 clusters, *n* = 1588 students) and a waitlist control (usual school provision; 11 clusters, *n* = 1892 children)). The trial statistician (but not schools or researchers) remained blind to allocation status. The outcomes were as follows: student-reported victimization (primary outcome) and bullying perpetration; teacher-reported child behavior and emotional well-being; and school absenteeism (administrative records). Implementation fidelity was measured using teacher-completed online records (for class lessons) and independent researcher observations (for school-wide elements). Outcome analyses involved 11 intervention schools (*n* = 1578 children) and 10 control schools (*n* = 1636 children). There was no statistically significant effect on the primary outcome of child-reported victimization (adjusted intervention/control OR 0.76; 95% CI 0.55 to 1.06; *p* = 0.11) or on the secondary outcomes. The impact on victimization was not moderated by child gender, age, or victimization status at baseline. Lesson adherence was good but exposure (lesson length) was lower than the recommended amount, and there was considerable variability in the implementation of whole school elements. The trial found insufficient evidence to conclude that KiVa had an effect on the primary outcome. A larger trial of KiVa in the UK is warranted, however, with attention to issues regarding implementation fidelity. Trial registration: Current Controlled Trials ISRCTN23999021 Date 10-6-13

**Electronic supplementary material:**

The online version of this article (10.1007/s11121-020-01103-9) contains supplementary material, which is available to authorized users.

## Introduction

Bullying refers to verbal, physical, or psychological aggression that is repeated over time and intended to cause harm or distress to the victims who are unable to defend themselves (Olweus 1992; Farrington 1993; Centers for Disease Control and Prevention 2014). It affects a large proportion of children. For example, a survey involving over 580,000 children aged 11, 13, and 15 years from 33 countries (31 European, two North American), reported that 29% of children were “occasional victims” (bullied at school once in the past couple of months) and 11% were “chronic victims” (bullied at least two or three times in the past couple of months) (Chester et al. 2015).

Victimization, or being bullied, is associated with psychological distress and carries numerous detrimental consequences that can persist into adulthood (Arseneault 2018), including depression (Ttofi et al. 2011a; Bowes et al. 2015); anxiety (Stapinski et al. 2014); self-harm (Fisher et al. 2012); suicidal ideation and suicide (Ttofi et al. 2011a); offending (Ttofi et al. 2011b); and high-risk health behaviors, such as drinking, smoking, and substance abuse (Vieno et al. 2011; Ttofi et al. 2016). It has also been associated with increased school absence (Brown et al. 2011), poorer educational attainment (Nakamoto and Schwartz 2010), lower lifetime earnings (Knapp et al. 2011), and greater use of mental health services (Evans-Lacko et al. 2017). An analysis of British birth cohort data shows that bullying in childhood also has adverse economic consequences at the individual and societal levels for men and women at age 50 (Brimblecombe et al. 2018). These include a lower likelihood of being employed or having accumulated wealth in the form of savings or home-ownership, and, for those who are frequently bullied, higher employment-related costs for men (loss of human capital) and higher health service costs for women.

For these reasons, it is important to address bullying. Targeted interventions concentrating solely at the level of the bully and/or the victim have had little success in reducing bullying (Vreeman and Carroll 2007; Rigby 2012) whereas multiple level whole school approaches have demonstrated significant effectiveness in reducing bullying behavior (Vreeman and Carroll 2007; Farrington and Ttofi 2009). A recent comprehensive meta-analysis involving 100 evaluations of whole school and targeted school-based anti-bullying programs found that, on average, bullying perpetration reduced by 19–20% and victimization by 15–16%, although there was significant variation between countries and programs (Gaffney et al. 2019).

### The KiVa Program

KiVa is a school-wide evidence-based program developed in Finland for children aged 7 to 15 years. Its primary focus is on changing the role of bystanders (fellow students who witness bullying events) as a means to prevent and stop bullying in schools. The program teaches children to recognize bullying and how to respond if they see bullying occur. It is based on research showing that bullies tend to behave aggressively to attain higher status and are reinforced by onlookers’ apathy or encouragement, and that when bystanders do intervene bullying tends to stop (Salmivalli et al. 2011). KiVa includes universal elements delivered at the school and class levels, and indicated elements that are used when bullying occurs.

The first randomized controlled trial (RCT) of KiVa, involving over 8000 children aged 9–12 years in 78 schools in Finland, found that it was effective for reducing self-reported victimization (intervention/control odds ratio (OR) 0.68) and bullying perpetration (OR 0.82) (Kärnä et al. 2011). Effects were slightly stronger on peer-reported measures (0.55 and 0.78 respectively). The positive effects on self-report measures were seen across all types of victimization, including verbal, physical, racist, sexual, and cyberbullying (Salmivalli et al. 2011). The same study found that KiVa reduced participants’ internalizing problems and improved their peer-group perceptions, with changes in anxiety, depression, and positive peer perceptions predicted by reduced victimization (Williford et al. 2012). A non-randomized evaluation of the national roll-out of KiVa in Finland using self-report measures also demonstrated positive effects, albeit smaller in size than in the trial: intervention/control OR of 0.82 for victimization and 0.85 for bullying (Kärnä et al. 2011). A second trial, also in Finland and involving both younger (6–9 years) and older (12–15 years) children, concluded that the effects of KiVa are larger and more consistent in elementary rather than lower secondary schools (Kärnä et al. 2013). An analysis of self-report data from both trials also showed positive effects on both cyberbullying (conditional on age) and cybervictimization (Williford et al. 2013). A recent analysis found that, based on the Finnish trials, the OR of being bullied in intervention versus control schools ranged from 0.55 to 0.88 and that the weighted mean treatment effect of KiVa corresponds to a relative risk of being bullied in a KiVa school compared with a status quo school of 0.58 (suggesting that it was lower in KiVa schools) (Persson et al. 2018).

Since then, an RCT in Italy, one of the first to explore the program’s transportability, involved children in two age cohorts (mean ages 8.9 and 10.9 years respectively) and a version of KiVa subjected to mostly surface program adaptations. It found small-to-medium effect sizes for continuous measures of bullying and victimization (Cohen’s *d* = 0.21 to 0.38), and supported hypothesized mechanisms of change, such as pro-victim empathy and reduced pro-bullying attitudes (Nocentini and Menesini 2016). However, on binary measures (of the kind used in previous studies of KiVa), there was a statistically significant reduction in victimization for the younger age cohort only (OR 0.52) and no significant effect for bullying in either age cohort.

### KiVa in Wales

In Wales, UK, local education authorities (LEAs) and governing bodies of maintained schools have a legal duty to safeguard and promote the well-being of all students, which includes a responsibility to tackle bullying (Welsh Government 2015). Schools must have an anti-bullying policy that sets out procedures for recording bullying incidents, investigating and dealing with incidents, supporting victims, and disciplining bullies (Estyn 2014). In the first comprehensive national survey in Wales of the prevalence and incidence of school bullying, 32% of Year 6 students (aged 10–11) reported that they had been bullied in the last 2 months, rising to 47% in the last year (Welsh Assembly Government 2010). A small pre-post pilot study of KiVa with 17 schools (14 in Wales, 3 in a neighboring county in England) in the academic year 2012–2013 (Hutchings and Clarkson 2015) found statistically significant reductions in self-reported victimization (16 to 9%) and bullying (6 to 2%) after 9 months (one academic year) of implementation (Clarkson 2015).

### The Present Study

The present study aimed to test the effectiveness of KiVa, measure the fidelity of its implementation, find out what teachers thought of the program (likes and dislikes, facilitators of and barriers to implementation), examine factors predicted to affect the scalability of the program, and calculate delivery costs (see Clarkson et al. 2016 for the trial protocol). This paper focuses on effectiveness and fidelity, with qualitative results regarding implementation reported elsewhere (DSRU et al. 2016). The effectiveness objectives were to evaluate whether KiVa: reduces student-reported victimization (primary outcome) and bullying perpetration; improves children’s emotional well-being; impacts positively on other aspects of children’s social and emotional well-being; and reduces school absenteeism. All outcomes are at the individual participant level. The fidelity objectives were to describe how well the class lessons and whole school elements were implemented. It was hypothesized that, relative to students in control schools, students in intervention schools would improve on all outcomes measured.

## Methods

### Trial Design

This study is a two-arm, waitlist control, pragmatic, parallel group cluster randomized controlled trial with a 1:1 allocation ratio. A cluster trial was necessary because KiVa is a whole school intervention. Schools were recruited in the middle of the 2012/13 academic year, with outcomes measured at the end of the 2013/14 academic year. Each school represents one cluster.

### Participants

All mainstream state-maintained primary schools in Wales were eligible for the study and invited to two half-day conferences in South Wales and North Wales respectively (March 2013) where we provided information on the following: KiVa and research on its effectiveness; the training, implementation, and support package; and the nature of the proposed evaluation. Participation was offered on a first-come-first-served basis to schools that attended a conference and confirmed, in writing, their commitment to (i) deliver the curriculum to all Key Stage (KS) 2 students (if randomized to the intervention arm) and (ii) participate in the evaluation. (KS2 refers to the 4 years of schooling when children are in Years 3 to 6 and aged 7 to 11 years.) School recruitment was completed by the end of April 2013. Students in recruited schools were eligible if they were in Years 2, 3, 4, and 5 (equivalent to US school grades 1 to 4; aged 6–10 years) in the 2012/13 academic year.

The incentives for school participation were free school materials, training, and KiVa registration for 2 years (the intervention schools were able to implement KiVa for a further year beyond the trial and the waitlist control schools were also able to implement KiVa for 2 years post-trial). No adverse consequences (e.g., loss of resources or money, or negative publicity) were foreseen for schools that might discontinue the intervention or deviate from the protocol. The proportion of children leaving schools or being absent at the time of the follow-up assessment was estimated as unlikely to be more than 10%.

### Sample Size

The aim was to randomize 10 schools (clusters) to each of the intervention and control arms (20 schools altogether) and recruit all children from Years 2 to 5 (6–10 years), following them up until they were in Years 3 to 6. Assuming unequal cluster sizes, and means of 1.25 classes in each year group and 25 children per class, it was estimated that there would be 125 eligible children in each school. Based on a 95% consent rate and a 10% drop-out rate, we anticipated that 1070 children would provide follow-up data in each trial arm at 12 months post-baseline (2140 children in total). The percentage of victimized children, the primary outcome, was previously estimated to be 16% (Clarkson 2015). With an assumed intra-cluster (intra-school) correlation coefficient of 0.025 (Farrington and Ttofi 2009) and mean cluster size of 107, our planned sample size was calculated to be large enough to detect a halving from 16 to 8% in the percentage of victimized children (equivalent to an OR of 0.46) with just over 80% power (81.6%) at the 5% (2-sided) level of significance.

### Randomization

Schools (clusters) were randomly allocated on a 1:1 basis to the intervention and control conditions. Randomization was carried out by an independent registered trials unit at Bangor University (the North Wales Organisation for Randomised Trials (NWORTH)). Complete list randomization using the dynamic adaptive algorithm (Russell et al. 2011) was implemented by a validated computer system, with stratification by size of school (“large” versus “small” split by the median) and proportion of children eligible for free school meals (“high” versus “low” split by the median). Researchers were unable to remain blind to school allocation, as the implementation evaluation was undertaken with schools when they were delivering the program. However, the trial statistician was blind to allocation status and a statistical analysis plan was written in advance of the analysis. Researchers informed schools of their assignment (intervention or control arm) in May 2013. Individual participants (students) were included in clusters (schools) by virtue of being in the relevant year group of a given school.

### Intervention

The *universal* element of KiVa comprises three curriculum units for children aged 7 to 9 (Unit 1), 10 to 12 (Unit 2), and 13 to 15 years (Unit 3) respectively. Units 1 and 2 were used in the Wales trial. Each contains 10 × 90-min lessons to be delivered monthly over a full academic year (September to July, 39 weeks), although they can also be delivered as 20 × 45-min lessons fortnightly over the same period. Lessons include film clips, group discussions and exercises. Additional universal elements are online games (to be played at home or at school), posters in the school building, and high-visibility vests for staff to wear in the playground during breaks to remind children they are in a KiVa school.

The *indicated* element involves school staff applying a standard protocol to address confirmed cases of bullying. A member (or members) of the KiVa team meets with the bullied victim and perpetrator(s) separately. The discussion with the perpetrator can be approached in one of two ways (at the school’s discretion). In the *confrontational* approach, the KiVa team refers to the perpetrator’s role in the bullying incident explicitly, before asking them to agree to a plan to address the problem. In the *non-confrontational* approach, the KiVa team explains that the victim is having a difficult time and asks the perpetrator to commit to helping to solve the problem. High-status peers nominated by the victim and recruited by the class teacher are encouraged to befriend and support the victim. A follow-up discussion with both victim and bully (or bullies) is held 2 weeks later to see if the bullying has stopped, and, if necessary, to repeat the process or move to other sanctions.

Intervention delivery began at the start of the school year (September 2013) and lasted until the summer term. Training was provided in the summer term prior to this (June/July 2013) by accredited KiVa trainers (authors JH and SC). Two members of the teaching/management team from each school were required to attend the one-day training. Follow-up school-based training was delivered to all school staff at the end of the school day. The intention was that KS2 class teachers would then teach the KiVa curriculum. Support and feedback sessions and a helpline were provided to assist with staff queries and improve school adherence to the intervention protocol.

### Control

Control schools were asked to continue with their usual practices in line with their bullying policy, while waiting 12 months to implement KiVa. Personal and Social Education (PSE) is an essential element of the basic curriculum for all students at maintained schools in Wales (Welsh Assembly Government 2008). The PSE curriculum aims to develop and explore the students’ values and attitudes, equip them to live safe and healthy lives, promote self-respect, celebrate diversity, and empower participation in school and community life as responsible citizens. Control schools were asked to continue to use their existing plan for covering the PSE curriculum. Schools use various strategies to prevent or address bullying and improve social interactions, such as peer support/mentoring schemes. The trial used a waitlist control design and KiVa was implemented in the control schools after the end of the trial (starting in September 2014).

### Measures

The primary outcome is student self-reported victimization, occurring at least twice a month in the last couple of months. Both victimization and one secondary outcome, student self-reported bullying perpetration, were measured using the Bully/Victim Questionnaire (BVQ) (Olweus 1996), which is part of the KiVa student online survey (Kärnä et al. 2011) completed by study participants. The global items: “How often have you been bullied at school in the last couple of months?” and “How often have you bullied others at school in the last few months?” were used to measure victimization and bullying, respectively. Students were asked to respond to both items on a 5-point scale (0, “not at all”; 1, “once or twice”; 2, “2 or 3 times a month”; 3, “about once a week”; 4, “several times a week”). Each item was dichotomized for analysis so that those scoring 2 to 4 were classified as victimized/bullied others and those scoring 0 or 1 as not victimized/did not bully others. This conceptual categorization (bullying concerns *repeated* acts) is supported by empirical research showing that there are large and highly significant differences between these groups on internalizing problems (for victims) and externalizing problems (for bullies) (Solberg and Olweus 2003). Intervention schools were trained in survey implementation during their KiVa training and control schools received written information about survey implementation which requires that children are reminded of the definition of bullying before each question. No monitoring of survey implementation was undertaken by the research team.

In order to measure children’s social and emotional well-being (also secondary outcomes), the teacher-reported Strengths and Difficulties Questionnaire (SDQ) (Goodman 1997, 1999) was administered at baseline and 12-month follow-up. It is a 25-item measure widely used in developmental, social, clinical, and educational studies to measure children’s mental health. The teacher version can be used for children aged 4 to 17 years. It comprises five subscales (5 items each) assessing hyperactivity, conduct, emotional difficulties, peer relations, and pro-social behavior, respectively, over the past 6 months. There are three response options for each item (0, “not true”; 1, “somewhat true”; 2, “certainly true”). For each subscale, the score can range from 0 to 10; a higher score indicates more problems for all subscales apart from the pro-social subscale, for which a higher score indicates more pro-social behavior. The “total difficulties score” is calculated by summing the scores for the first four subscales (total score ranges from 0 to 40, with higher scores indicating greater problems).

The SDQ also has a brief “Impact supplement” which starts with a single question about whether the child has difficulties with emotions, concentration, behavior, or being able to get on with other people (response set: “No”; “Yes—minor difficulties”; “Yes—definite difficulties”; “Yes—severe difficulties”). If the answer is “Yes,” there are four additional questions, focusing (in the teacher version) on the following: chronicity, or duration; distress to the child; impact on the child’s everyday life in terms of peer relations and classroom learning respectively; and burden to the teacher or class as a whole. The teacher-report impact score is calculated by summing responses to three items, namely (i) whether the difficulties upset or distress the child, and impact on (ii) peer relations and (iii) classroom learning, with the total score ranging from 0 to 6, where higher scores indicate greater impact.

A review (Stone et al. 2010) of the psychometric properties of the teacher-completed SDQ, examining 26 studies involving teachers of children aged between 4 and 12 years, estimated the overall Cronbach’s alpha of inter-item reliability to be 0.73 for the emotional symptoms subscale, 0.82 for pro-social behavior, 0.70 for conduct problems, 0.63 for peer problems, 0.82 for the total difficulties score, and 0.85 for the impact score. The same paper reported that the pooled test-retest reliability correlation from six studies was also high for the total difficulties score (Pearson’s correlation (*r*) = 0.84) and the impact score (*r* = 0.68).

Schools were asked to provide records of authorized and unauthorized half-day absences at the student level for participating students in the study for the academic years 2012–2013 (baseline) and 2013–2014 (12-month follow-up). These data are routinely collected by schools for all students as a legal requirement. Schools were asked to provide the anonymized attendance data linked to the KiVa identification numbers to protect student anonymity.

### Data Collection

Baseline data were collected via the school-administered student online KiVa survey (classroom or computer lab) and via online teacher surveys in intervention and control schools in June/July 2013 for students in Years 2 to 5 (i.e., about to enter KS2 Years 3 to 6). Data on the same measures were collected at 12 months post-baseline (June/July 2014) for students coming to the end of Years 3, 4, 5, and 6. In most cases, this meant that follow-up SDQs were completed by different teachers as students had moved to a different class. Ethnicity, free school meals eligibility, and special education needs (SEN) status (for baseline) and absence data (for the academic years 2012–2013 and 2013–2014) were collected in Autumn 2015.

### Fidelity

Teachers used online record books to document the following: time spent preparing each lesson; time spent delivering each lesson; which parts of the lesson were delivered; their view on lesson content suitability; and the proportion of students engaging positively in the lesson. They were encouraged to complete these immediately following the relevant lesson. In accordance with previous research on the fidelity of delivering KiVa lessons (Haataja et al. 2014), the analysis focused on adherence (to lesson content), exposure (lesson length), and quality (using time spent preparing lessons as a proxy). Lesson adherence was calculated as the proportion of tasks delivered for each lesson averaged over the 10 lessons (expressed as a percentage). Lesson length was calculated as the number of minutes used for teaching lesson content averaged across the lessons a teacher is reported to have delivered. Time spent preparing the lessons was calculated by averaging the reported number of minutes across the lessons delivered by a teacher.

School-wide program implementation was assessed by independent observation (one per school) in May/June 2014. Two members of the research team who understood the main aims, theory, and components of the intervention scored each of the following seven items on a 3-point scale (0, “not true”; 1, “somewhat true”; 2, “certainly true”): the visibility of KiVa materials in the school; the extent to which the head teacher, playtime supervisors, a KS2 teacher (or the KiVa team lead) and KS2 students could talk knowledgably about the program (conditional on respondent role, the criteria covered program ethos, constituent activities, process for addressing reports of bullying, membership of the KiVa team, lesson and online game content, and the respondent's own role in the program); and evidence of a KiVa team logbook being used to record bullying incidents and how they were dealt with. Item scores were summed to give an overall score for each school (range 0 to 14), where a higher score indicated stronger school-wide implementation. Since each researcher visited different schools, they discussed their ratings to ensure consistency.

### Analysis

The analysis estimated differences at 12-month follow-up between the two trial arms, adjusting for baseline data. Baseline characteristics of the schools and students were summarized separately for each trial arm. Comparison of outcomes at follow-up was based on the intention-to-treat (ITT) principle with schools (clusters) and students analyzed according to the trial arm they were allocated to, irrespective of the level of intervention actually received. Comparisons between the trial arms were carried out after using multiple imputation to impute data for participants with missing values. Binary outcomes were compared between trial arms using marginal logistic regression models using Generalized Estimating Equations (GEEs) with information sandwich (“robust”) estimates of standard error assuming an exchangeable correlation structure. An odds ratio less than one indicates that the odds of bullying/victimization is lower in the intervention arm than in the control arm. The absenteeism rate was compared between trials arms using the GEE method specifying the Poisson distribution and log link function. A rate ratio less than one indicates that the rate of absenteeism is lower in the intervention arm than in the control arm. Continuous outcomes were compared using random effects linear regression. All methods allow for correlation of outcomes within schools (clusters). Analyses were adjusted for the following: the baseline score for the outcome; the school-level variables of school size and free school meals eligibility at baseline; and child gender, age, special education needs status, and free school meals status. Stata 13.1 was used for the analyses using the *mi impute* and *mi estimate* commands to generate 20 imputed datasets and analyze these, respectively.

## Results

**Fig. 1:**
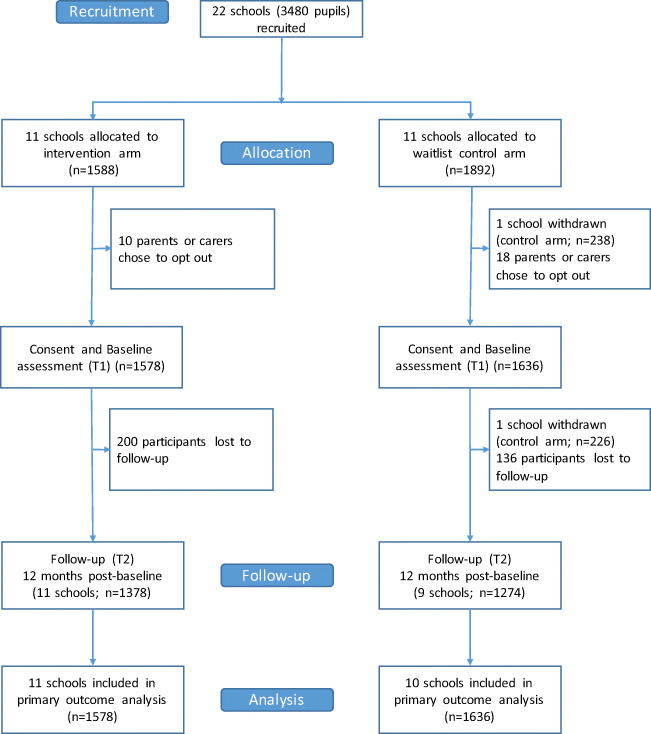
CONSORT diagram

In total, 22 schools from across Wales were recruited for the trial (22 schools applied and met the criteria, so rather than reject two, and since capacity was available, it was decided to allow all of them to take part). Of these, 11 were randomized to the intervention arm and 11 to the control arm (Fig. 1). Based on the median split for the stratification variables, there were five “large” schools in the intervention arm and six in the control arm, and five schools with a “high” proportion of free school meals in the intervention arm and six in the control arm. Two control schools withdrew during the first year—one before and one after baseline data collection respectively. Table 1 shows the baseline characteristics of the two trial arms for the 21 schools that provided baseline data. At baseline, there were 3214 students in the sample overall—1578 in the intervention (I) arm and 1636 in the control (C) arm. The age and gender split in both arms were broadly even, as was socio-economic status (indicated by eligibility for free school meals). The proportion of children from minority ethnic groups was marginally higher in the intervention arm (10.2% I versus 5.2% C), while the control arm had a higher proportion of children with special educational needs. The rate of bullying victimization was higher in the control arm (20.2% I versus 26.0% C), as was the rate of bullying perpetration (6.9% I versus 8.7% C). Baseline data on the SDQ and absenteeism show only very marginal differences between the trial arms.

**Table 1:** Baseline characteristics of children in the intervention and control arms

		Intervention (*N* = 1578)	Control (*N* = 1636)
Gender	Male, *n* (%)	711 (45.1)	725 (44.3)
	Female, *n* (%)	717 (45.4)	684 (41.8)
	Missing, *n* (%)	150 (9.5)	227 (13.9)
Age in years	Mean (SD)	8.8 (1.1)	8.9 (1.2)
Eligible for free school meals (FSM)	Yes, *n* (%)	237 (15.0)	220 (13.4)
	No, *n* (%)	1116 (70.7)	931 (56.9)
	Missing, *n* (%)	225 (14.3)	485 (29.6)
Special educational needs (SEN) status	No SEN, *n* (%)	1025 (65.0)	756 (46.2)
	School Action, *n* (%)	180 (11.4)	220 (13.4)
	School Action Plus, *n* (%)	121 (7.7)	171 (10.5)
	Statement, *n* (%)	27 (1.7)	4 (0.2)
	Missing, *n* (%)	225 (14.3)	485 (29.6)
Ethnicity	White, *n* (%)	1176 (74.5)	1018 (62.2)
	Asian, *n* (%)	78 (4.9)	15 (0.9)
	Black, *n* (%)	18 (1.1)	6 (0.4)
	Mixed, *n* (%)	65 (4.1)	39 (2.3)
	Other, *n* (%)	2 (0.1)	26 (1.6)
	Refused, *n* (%)	6 (0.4)	10 (0.6)
	Missing, *n* (%)	233 (14.8)	522 (31.9)
Bullying victim	Yes, *n* (%)	318 (20.2)	426 (26.0)
	No, *n* (%)	1097 (69.5)	1035 (63.3)
	Missing, *n* (%)	163 (10.3)	175 (10.7)
Bullying perpetrator	Yes, *n* (%)	109 (6.9)	142 (8.7)
	No, *n* (%)	1306 (82.8)	1319 (80.6)
	Missing, *n* (%)	163 (10.3)	175 (10.7)
SDQ scores^#^	Emotional symptoms	1.5 (2.1)	1.4 (2.0)
	Peer relationship problems	1.1 (1.7)	1.1 (1.6)
	Conduct problems	0.9 (1.7)	0.9 (1.7)
	Prosocial behavior	8.2 (2.3)	8.1 (2.4)
	SDQ total difficulties	6.3 (6.2)	6.3 (6.3)
	Impact	0.5 (1.2)	0.4 (1.1)

^#^Mean (SD)

Individual level categorical baseline characteristics (all schools except for the one school that withdrew before baseline)

Sample size for age in years was 1423 for the intervention arm and 1394 for the control arm

Sample size for SDQ scores was 1425 for the intervention arm and 1407 (1406 for Impact) for the control arm

### Outcomes

The data were analyzed to look at the impact of KiVa on outcomes. These results are based on the 21 schools (11 intervention, 10 control) that provided baseline data. Rates of follow-up were reasonably high for outcome measures: bullying questionnaire (87% I, 75% C); SDQ (89% I, 81% C); and attendance (91% I, 92% C). Missing values were imputed.

There were no statistically significant effects on either the primary outcome measure of child-reported victimization (adjusted odds ratio (OR) 0.76; 95% CI 0.55 to 1.06; *p* = 0.11) or the secondary outcome measures of child-reported bullying perpetration (adjusted OR 0.89; 95% CI 0.61 to 1.28; *p* = 0.51) and teacher-rated child emotional difficulties (adjusted mean difference − 0.008; 95% CI − 0.4 to 0.4, *p* = 0.97) (Table 2).

**Table 2:** Outcomes by trial arm status

Outcome	Intervention (I)	Control (C)	Unadjusted		Adjusted		
	Mean (SD)/(%)	Mean (SD)/(%)	Mean diff/OR	ICC	Mean diff/OR	95% CI	*p* value
Primary outcome							
Victimization	14.6%	19.8%	0.73	0.019	0.76	0.55 to 1.06	0.11
Secondary outcomes							
Bullying perpetration	5.1%	6.7%	0.82	0.009	0.89	0.61 to 1.28	0.51
SDQ Emotional symptoms score	1.3 (2.1)	1.4 (2.1)	− 0.006	0.097	− 0.008	− 0.4 to 0.4	0.97
SDQ Conduct problems score	0.9 (1.6)	1.0 (1.9)	− 0.003	0.054	0.001	− 0.2 to 0.2	0.99
SDQ Peer relationship problems score	1.0 (1.6)	1.0 (1.7)	0.03	0.049	0.05	− 0.2 to 0.3	0.63
SDQ Prosocial behavior score	8.2 (2.2)	8.3 (2.2)	− 0.08	0.032	− 0.2	− 0.5 to 0.1	0.19
SDQ Total difficulties score	5.6 (6.2)	6.1 (6.8)	− 0.3	0.082	− 0.1	− 1.1 to 0.8	0.76
SDQ Impact score status	16.7%	19.6%	0.92	0.038	0.92	0.62 to 1.37	0.68

*SD* standard deviation, *OR* odds ratio

Sample size 1578 in the intervention arm and 1636 in the control arm

Intra-cluster (intra-school) correlation coefficient (ICC) estimates are from analyses of participants with complete data

There were also no statistically significant effects on the other secondary outcomes, namely, teacher-rated child conduct problems, peer relationship problems, pro-social behavior and total difficulties, and absenteeism rates (Table 2). Children were absent from school for 23,714 of a total possible 486,153 half-days in the intervention arm and 17,613 of a total possible 351,858 half-days in the control arm. The absenteeism rate was 4.9 per 100 half-days per child in the intervention arm and 5.0 per 100 half-days in the control arm. The adjusted rate ratio for absenteeism was 1.04 (95% CI 0.95 to 1.14; *p* = 0.38).

### Moderators

There was little evidence that the effect of the intervention on victimization differed by gender (*p* value for test of interaction = 0.82), age (< 9 years vs. ≥ 9 years; *p* = 0.73) or between children who were and were not victimized at baseline (*p* = 0.94).

### Fidelity

**Fig. 2:**
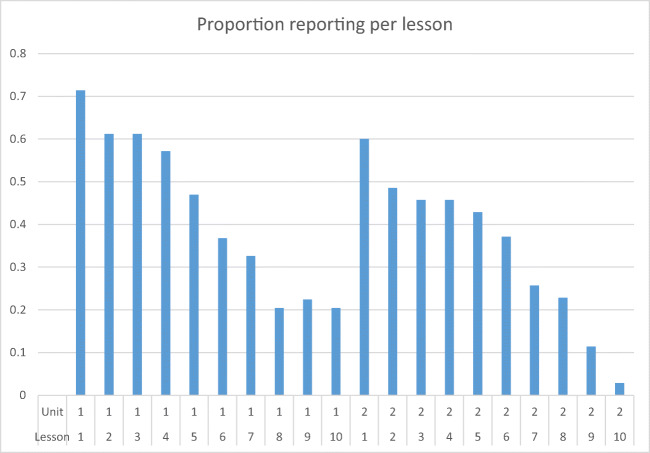
Proportion of completed lesson records for each lesson

Regarding fidelity, lesson records were completed for at least one of the 20 lessons (across two units) for 65 identifiable classes in the intervention arm (96% of classes), although reporting diminished over the course of units (Fig. 2). Thus, lesson records were missing for over half of many lessons (58% of data missing overall). For those lessons for which records were available, teachers reported delivering 90% of lesson components on average. The median preparation time per lesson was 20 min (interquartile range, 15 to 30) and the median delivery time per (full) lesson was 60 min (interquartile range, 45 to 90).

Visits were completed in all 11 intervention schools. The mean (M) total score for the school observation measure was 8.0 out of 14 (standard deviation (SD) = 2.2), and on average schools scored just above 1 out of 2.0 per item (M (SD) = 1.2 (0.3)) (Table 3). In general, schools scored higher on items concerning stakeholders’ knowledge of KiVa, with teachers (M (SD) = 1.6 (0.5)) and head teachers (M (SD) = 1.6 (0.5)) scoring highest (out of 2.0). Scores were lower for items concerning the implementation of whole school elements. Schools were fairly reliable in displaying KiVa posters (M (SD) = 1.3 (0.5)), with three schools displaying them in all communal areas, and all other schools displaying them in some but not all communal areas. However, only five schools provided evidence of keeping a KiVa team logbook, with an overall mean score less than one (M (SD) = 0.7 (0.9)), and the same number had school staff wearing the KiVa vests/tops during playtime (M (SD) = 0.7 (0.6)).

**Table 3:** Mean (SD) scores per item measured in school observations

Item	Mean (SD)
A KS2 teacher can talk fluently and knowledgably about KiVa	1.6 (0.5)
The head teacher can talk fluently and knowledgably about KiVa	1.6 (0.5)
KS2 students can talk fluently and knowledgably about KiVa	1.3 (0.5)
Playtime supervisors can talk fluently and knowledgably about KiVa	1.1 (0.3)
KiVa posters are visible in the school, including in communal areas	1.3 (0.5)
There is a KiVa team logbook with a record of bullying incidents reported and how they were handled	0.7 (0.9)
Playtime supervisors wear the KiVa vests/tops during playtime	0.7 (0.6)

## Discussion

The KiVa intervention had no statistically significant effect on child-reported bullying victimization and perpetration. Nor was there an effect on teacher-reported child emotional and behavioral difficulties or absenteeism rates. The results may be generalized to other settings in which there is a requirement to address bullying in schools and where social-emotional lessons are taught.

The first possible reason for the lack of effect concerns implementation fidelity. For reasons outlined below, it was not possible to undertake a meaningful analysis of the relationship between fidelity and outcomes, and other evidence to support this hypothesis is mixed. Self-completed teacher lesson records suggest that adherence was good where reported—indeed, higher than reported previously (Haataja et al. 2014)—although given the large amount of missing data, it is plausible that this overstates the reality. Regarding dosage, average lesson delivery times were substantially less than the recommended 90 min and the mean (79 min) found in a study in which lesson duration was significantly correlated with lesson adherence (Haataja et al. 2014). There was considerable variability in the extent to which the posters and especially the vests and incident logbook were implemented. Interviews with school staff reveal that while the program was broadly well received by teachers (and parents and children), challenges with implementation arguably undermined fidelity. Examples included teachers omitting program content to fit KiVa into an already packed curriculum, and IT issues preventing children from playing the online games (DSRU et al. 2016). It might be that schools need more intensive and responsive implementation support than was offered (support in the Finnish and Italian trials was arguably more intensive). Future studies should deliberately vary the nature and extent of such support in order to establish what is optimal.

A second possible reason relates to administration of the annual student online survey. Largely anecdotal evidence indicates that this was highly variable across the schools. Moreover, the administration details included the need to remind children that bullying involves perpetration by a higher status individual or individuals, and is both deliberate and repeated. Training on survey administration was given to intervention schools prior to baseline data collection during training in intervention delivery, whereas control schools only had the written guidance at this point. It is therefore possible that baseline survey administration in control schools was different than in intervention schools, potentially contributing to higher reported rates of victimization and bullying if students were not thinking of the specific definition of bullying when responding. Although rates of both victimization and bulling were higher in the control arm at baseline, we do not have empirical evidence of whether survey administration was different in intervention and control schools at baseline, or, if it was, whether and how it influenced survey responses. Future research should take care to ensure that survey administration is identical in both conditions.

A third potential explanation relates to the nature and quality of other bullying-related provision. Control schools continued to deliver regular PSE lessons, whereas in intervention schools, it is possible that KiVa lessons replaced them (the KS2 KiVa program maps onto the PSE curriculum, covering over 50% of it; intervention schools received a copy of this mapping to enable them to incorporate the KiVa lessons into their school PSE plan). While data on the delivery of non-KiVa strategies or programs were not collected in the present trial, future studies should do this systematically, particularly since doing so is rare in trials of bullying programs, including KiVa.

The study has several strengths, notably the randomized design, the use of tried-and-tested measures, and the collection of data on different elements of the fidelity of classroom lessons and school-wide implementation. However, the study also has limitations. First is the large amount of missing data on lesson implementation; in future studies, investigators should send teachers regular reminders to boost completion rates. Second, we were unable to analyze the relationship between fidelity and outcomes because we do not know which classes students were in when KiVa was delivered; class IDs were assigned at baseline, the academic year before KiVa started, and students may have been in different groupings in the new academic year. Third, there appeared to be variation in how the student survey was implemented, although its impact on results is unclear. Fourth, it is not clear what non-KiVa bullying prevention activities were delivered by schools in either trial arm. Fifth, we did not investigate program impact on different types of bullying, and last, data on victimization and perpetration were only collected from children (not peers or teachers).

## Conclusions

A trial of KiVa involving data on over 3000 children from 21 primary schools in Wales found insufficient evidence to conclude that the program had an effect on the primary outcome, namely child-reported rates of bullying victimization. There were no effects on bullying perpetration, teacher-reported child behavioral and emotional difficulties or absenteeism rates. A new trial^1^ of KiVa will explore its impact in a wider UK context, attending to problems with fidelity identified in the current study and recording carefully all bullying-related activities undertaken in intervention and control schools.

## Electronic supplementary material

ESM 1 (DOCX 31 kb)

## Footnotes

1 https://fundingawards.nihr.ac.uk/award/17/92/11

## References

[CR1] Arseneault L (2018). Annual research review: The persistent and pervasive impact of being bullied in childhood and adolescence: Implications for policy and practice. Journal of Child Psychology and Psychiatry.

[CR2] Bowes, L., Joinson, C., Wolke, D., & Lewis, G. (2015). Peer victimisation during adolescence and its impact on depression in early adulthood: Prospective cohort study in the United Kingdom. *BMJ, 350*:h2469. 10.1136/bmj.h2469 PMC4452929 26037951

[CR3] Brimblecombe N, Evans-Lacko S, Knapp M, King D, Takizawa R, Maughan B, Arseneault L (2018). Long term economic impact associated with childhood bullying victimisation. Social Science & Medicine.

[CR4] Brown V, Clery E, Ferguson C (2011). Estimating the prevalence of young people absent from school due to bullying.

[CR5] Centers for Disease Control and Prevention (2014). Bullying surveillance among school-aged children: Uniform definitions and recommended data elements.

[CR6] Chester KL, Callaghan M, Cosma A, Donnelly P, Craig W, Walsh S, Molcho M (2015). Cross-national time trends in bullying victimization in 33 countries among children aged 11, 13 and 15 from 2002 to 2010. European Journal of Public Health.

[CR7] Clarkson S (2015). KiVa anti-bullying programme. Paper presented at the presentation at the Centre of Evidence Based Early Interventions Conference, 5th march.

[CR8] Clarkson S, Axford N, Berry V, Edwards RT, Bjornstad G, Wrigley Z (2016). Effectiveness and micro-costing of the KiVa school-based bullying prevention programme in Wales: Study protocol for a pragmatic definitive parallel group cluster randomised controlled trial. BMC Public Health.

[CR9] DSRU (Dartington Social Research Unit), Centre for Evidence-based Early Intervention (Bangor University), Centre for Health Economics and Medicnes Evaluation (Bangor University), & NIHR CLAHRC South West Peninsula (PenCLAHRC) (University of Exeter) (2016). An Evaluation of the Implementation, Impact and Potential for Scale-up of the KiVa Anti-bullying Programme in Wales: A Report for the Big Lottery: unpublished report.

[CR10] Estyn (2014). Action on bullying: A review of the effectiveness of action taken by schools to address bullying on the grounds of students’ protected characteristics.

[CR11] Evans-Lacko S, Takizawa R, Brimblecombe N, King D, Knapp M, Maughan B, Arseneault L (2017). Childhood bullying victimization is associated with use of mental health services over five decades: A longitudinal nationally representative cohort study. Psychological Medicine.

[CR12] Farrington DP (1993). Understanding and preventing bullying. Crime and Justice.

[CR13] Farrington, D. P., & Ttofi, M. M. (2009). School-based programs to reduce bullying and victimization. *Campbell Systematic Reviews, 5*(1), i-148. 10.1002/cl2.1143 PMC8356322 37131921

[CR14] Fisher HL, Moffitt TE, Houts RM, Belsky DW, Arseneault L, Caspi A (2012). Bullying victimisation and risk of self harm in early adolescence: Longitudinal cohort study. BMJ.

[CR15] Gaffney H, Farrington DP, Ttofi MM (2019). Examining the effectiveness of school-bullying intervention programs globally: A meta-analysis. International Journal of Bullying Prevention.

[CR16] Goodman R (1997). The Strengths and Difficulties Questionnaire: A research note. Journal of Child Psychology and Psychiatry.

[CR17] Goodman R (1999). The extended version of the Strengths and Difficulties Questionnaire as a guide to child psychiatric caseness and consequent burden. Journal of Child Psychology and Psychiatry.

[CR18] Haataja A, Voeten M, Boulton AJ, Ahtola A, Poskiparta E, Salmivalli C (2014). The KiVa antibullying curriculum and outcome: Does fidelity matter?. Journal of School Psychology.

[CR19] Hutchings J, Clarkson S (2015). Introducing and piloting the KiVa bullying prevention programme in the UK. Educational & Child Psychology.

[CR20] Kärnä A, Voeten M, Little T, Poskiparta E, Alanen E, Salmivalli C (2011). Going to scale: A nonrandomized nationwide trial of the KiVa antibullying program for grades 1-9. Journal of Consulting and Clinical Psychology.

[CR21] Kärnä A, Voeten M, Little TD, Alanen E, Poskiparta E, Salmivalli C (2013). Effectiveness of the KiVa antibullying program: Grades 1-3 and 7-9. Journal of Educational Psychology.

[CR22] Knapp M, McDaid D, Parsonage M (2011). Mental health promotion and mental illness prevention: The economic case.

[CR23] Nakamoto J, Schwartz D (2010). Is peer victimization associated with academic achievement? A meta-analytic review. Social Development.

[CR24] Nocentini A, Menesini E (2016). KiVa anti-bullying program in Italy: Evidence of effectiveness in a randomized control trial. Prevention Science.

[CR25] Olweus D, Peters RD, McMahon RJ, Quinsey VL (1992). Bullying among school children: Intervention and prevention. Aggression and violence throughout the lifespan.

[CR26] Olweus, D. (1996). *The Revised Olweus Bully / Victim Questionnaire*. Bergen: University of Bergen, Research Center for Health Promotion (HEMIL Center).

[CR27] Persson M, Wennberg L, Beckman L, Salmivalli C, Svensson M (2018). The cost-effectiveness of the Kiva antibullying program: Results from a decision-analytic model. Prevention Science.

[CR28] Rigby K (2012). Bullying interventions in schools: Six basic approaches.

[CR29] Russell D, Hoare ZSJ, Whitaker R, Whitaker CJ, Russell IT (2011). Generalized method for adaptive randomization in clinical trials. Statistics in Medicine.

[CR30] Salmivalli C, Kärnä A, Poskiparta E (2011). Counteracting bullying in Finland: The KiVa program and its effects on different forms of being bullied. International Journal of Behavioral Development.

[CR31] Solberg ME, Olweus D (2003). Prevalence estimation of school bullying with the Olweus Bully/Victim Questionnaire. Aggressive Behavior.

[CR32] Stapinski LA, Bowes L, Wolke D, Pearson RM, Mahedy L, Button KS (2014). Peer victimization during adolescence and risk for anxiety disorders in adulthood: A prospective cohort study. Depression and Anxiety.

[CR33] Stone LL, Otten R, Engels RCME, Vermulst AA, Janssens JMAM (2010). Psychometric properties of the parent and teacher versions of the Strengths and Difficulties Questionnaire for 4- to 12-year-olds: A review. Clinical Child and Family Psychology Review.

[CR34] Ttofi MM, Farrington DP, Lösel F, Loeber R (2011). Do the victims of school bullies tend to become depressed later in life? A systematic review and meta-analysis of longitudinal studies. Journal of Aggression, Conflict and Peace Research.

[CR35] Ttofi MM, Farrington DP, Lösel F, Loeber R (2011). The predictive efficiency of school bullying versus later offending: A systematic/meta-analytic review of longitudinal studies. Criminal Behaviour and Mental Health.

[CR36] Ttofi MM, Farrington DP, Lösel F, Crago RV, Theodorakis N (2016). School bullying and drug use later in life: A meta-analytic investigation. School Psychology Quarterly.

[CR37] Vieno A, Gini G, Santinello M (2011). Different forms of bullying and their association to smoking and drinking behavior in Italian adolescents. Journal of School Health.

[CR38] Vreeman RC, Carroll AE (2007). A systematic review of school-based interventions to prevent bullying. Archives of Pediatrics & Adolescent Medicine.

[CR39] Welsh Assembly Government (2008). Personal and social education framework for 7 to 19-year olds in Wales.

[CR40] Welsh Assembly Government (2010). A survey into the prevalence and incidence of school bullying in Wales.

[CR41] Welsh Government (2015) *Keeping learners safe: The role of local authorities, governing bodies and proprietors of independent schools under the Education Act 2002*. Guidance document 158/2015. Cardiff: Welsh Government.

[CR42] Williford A, Boulton A, Noland B, Little TD, Kärnä A, Salmivalli C (2012). Effects of the KiVa anti-bullying program on adolescents’ depression, anxiety, and perception of peers. Journal of Abnormal Child Psychology.

[CR43] Williford A, Elledge LC, Boulton AJ, DePaolis KJ, Little TD, Salmivalli C (2013). Effects of the KiVa antibullying program on cyberbullying and cybervictimization frequency among Finnish youth. Journal of Clinical Child & Adolescent Psychology.

